# Responses of African Grasses in the Genus *Sporobolus* to Defoliation and Sodium Stress: Tradeoffs, Cross-Tolerance, or Independent Responses?

**DOI:** 10.3390/plants2040712

**Published:** 2013-11-08

**Authors:** Daniel M. Griffith, T. Michael Anderson

**Affiliations:** Department of Biology, Wake Forest University, Winston-Salem, NC 27109, USA; E-Mail: anderstm@wfu.edu

**Keywords:** sodium, defoliation, stress, stress interactions, salinity, congener comparison

## Abstract

In the Serengeti ecosystem of East Africa, grazing ungulates prefer areas with elevated grass Na, suggesting that some grasses tolerate both high soil Na and defoliation. We performed a factorial Na-by-defoliation greenhouse study with five abundant *Sporobolus* congeners to explore whether Serengeti grasses possess traits which: (i) confer tolerance to both Na and defoliation (cross-tolerance); (ii) display a tradeoff; or (iii) act independently in their tolerances. Our expectation was that related grasses would exhibit cross-tolerance when simultaneously subjected to Na and defoliation. Instead, we found that physiological tolerances and growth responses to Na and defoliation did not correlate but instead acted independently: species characterized by intense grazing in the field showed no growth or photosynthetic compensation for combined Na and defoliation. Additionally, in all but the highest Na dosage, mortality was higher when species were exposed to both Na and defoliation together. Across species, mortality rates were greater in short-statured species which occur on sodic soils in heavily grazed areas. Mortality among species was positively correlated with specific leaf area, specific root length, and relative growth rate, suggesting that rapidly growing species which invest in low cost tissues have higher rates of mortality when exposed to multiple stressors. We speculate that the prevalence of these species in areas of high Na and disturbance is explained by alternative strategies, such as high fecundity, a wide range of germination conditions, or further dispersal, to compensate for the lack of additional tolerance mechanisms.

## 1. Introduction

A primary goal of studying plant-herbivore interactions is to understand how plants cope with losing significant amounts of their biomass to herbivores. However, the loss of tissue to animals is but one of many “stressors” to which plants have evolved adaptations. Theory suggests that the evolution of plant adaptations to one particular stress comes at a cost to other adaptations [1,2]. As a result, adaptations are often characterized by tradeoffs: plants that are adapted to tolerate one set of conditions will be intolerant of other sets of conditions [3]. For example, Amazonian tree species specialized to grow on clay soils have higher growth rates than plants growing on sandy soil but are less resistant to herbivory [4,5]. In natural systems, which are inherently heterogeneous, tradeoffs among adaptations and tolerances are believed to promote coexistence among plant species [6,7,8].

Despite the theoretical importance of tradeoffs in explaining biological diversity, several processes can produce adaptations or tolerances that are positively correlated among species. First, the response of a plant to one stress can sometimes provide a benefit in tolerating other stresses (e.g., cross-tolerance) [9]. Tolerances can also be related because one is an evolutionary pre-requisite for another, or because a new functionality was added to a key adaptation over evolutionary time (e.g., exaptation) [10,11,12,13,14]. For example, many of the grazing tolerance traits common to many Poaceae may in fact be ancestral adaptations to drought conditions [15]. Lastly, it is possible for two tolerance traits to be separately adaptive but not conflicting in a significant way [16].

In many grazing ecosystems, persistence of grasses in high Na soils challenges the expectation that adaptation to abiotic stress should conflict with tolerance of tissue loss to herbivores [17,18]. This is because grazers seeking Na preferentially defoliate Na-accumulating plants [19,20]. Serengeti National Park (SNP), Tanzania, is characterized by gradients of soil Na due to volcanic ash, rainfall, and topography [21,22,23,24]. Grazing intensity increases in the Serengeti Plains where Na can also be high. For plants that coexist along these Na gradients, Na tolerance implies the ability to persist (grow, survive, or reproduce) in soils with low water potential while managing the specific effects of high exchangeable soil Na ions [25]. Moreover, in some Plains species such as *Sporobolus kentrophyllus*, defoliation increases root Na uptake rates—along with nitrogen, potassium, and phosphorus—indicating that rapid nutrient acquisition may be a general fitness benefit to heavily grazed plants [26]. Because of the attractiveness of Na as a forage nutrient for herbivores [27], plants that accumulate Na may experience higher levels of herbivory. This raises the possibility that plants are dually adapted to high soil Na and herbivory. Consequently, plant-herbivore interactions in grazing ecosystems present somewhat of a conundrum: plants must simultaneously tolerate biotic and abiotic plant stress, whereas theory predicts tradeoffs between major adaptations.

The primary objective of our research was to study the responses of five Serengeti *Sporobolus* species to soil Na addition and defoliation in order to explore the evolution of stress tolerance among species in a closely related clade of sympatric grasses. The experimental species included three species distributed in heavily grazed areas (*Sporobolus pellucidus*, *S. ioclados*, and *S. fimbriatus*) and two from areas of lesser grazing intensity (*S. pyramidalis*, and *S. consimilis*); these congeners show wide variation in traits associated with defoliation tolerance and have somewhat overlapping ranges in SNP [28,29,30,31,32]. In order to explore the potential mechanisms by which traits and tolerance might relate we examined the relative growth rate (RGR) and physiological responses of these five species to different soil Na concentrations, with and without clipping, in a 10 week greenhouse study. Replicates of each species were subjected to one of four levels of soil Na addition (0, 100, 200, and 400 mM Na) and were either defoliated or not. Defoliated plants had 50% of their aboveground biomass clipped with shears to apply comparable tissue loss across species. We measured and analyzed the RGR, leaf photosynthetic properties, and survivorship of plants as responses to these treatments. RGR was measured as the relative rate of growth over the entire study. Photosynthetic properties were measured two days and 14 days after application of treatment and included carbon assimilation (A_0_), stomatal conductance (G_s_), and evapotranspiration (E). Finally, we investigated the association between life-history strategies and the experimental mortality of each species by comparing functional trait patterns (e.g., specific leaf area or SLA) and survivorship among species.

## 2. Results

### 2.1. Growth Rate & Photosynthetic Responses

Irrespective of treatment, *S. pellucidus* had the highest overall RGR and *S. consimilis* the lowest. Across all species defoliation decreased RGR (Table 1). There was no interaction effect of Na and defoliation on RGR. Na treatment reduced RGR at 200 and 400 mM Na^+^ across species but increased RGR at 100 mM. In this experiment none of our species fully compensated for defoliated tissue.

**Table 1:** Regression coefficients (β) for linear mixed model results from five *Sporobolus* species treated with Na (four levels including 0 mM) and clipped in a full factorial experiment. The table shows the parameter estimates for all treatment levels for significant predictors (see Table A1). RGR = relative growth rate; A_o_ = net carbon assimilation; G_s_ = stomatal conductance; and E = transpiration.

Response	Na^+^ treatment (β's)			Defoliation (β's)	Na^+^ x Defoliation (β's)		
	100 mM	200 mM	400 mM	Clipped	Clipped	Clipped	Clipped
					100 mM	200 mM	400 mM
RGR	0.001	−0.004	−0.008	−0.01			
*Day 2*							
A_0_	1.09	−3.96	−9.11				
Gs					−0.021	−0.01	−0.029
E					−1.23	−0.67	−1.8
*Day 14*							
A_0_					−4.84	−7.44	0.52
Gs					−0.022	−0.026	−0.002
E					−1.59	−1.8	−0.16

**Figure 1:**
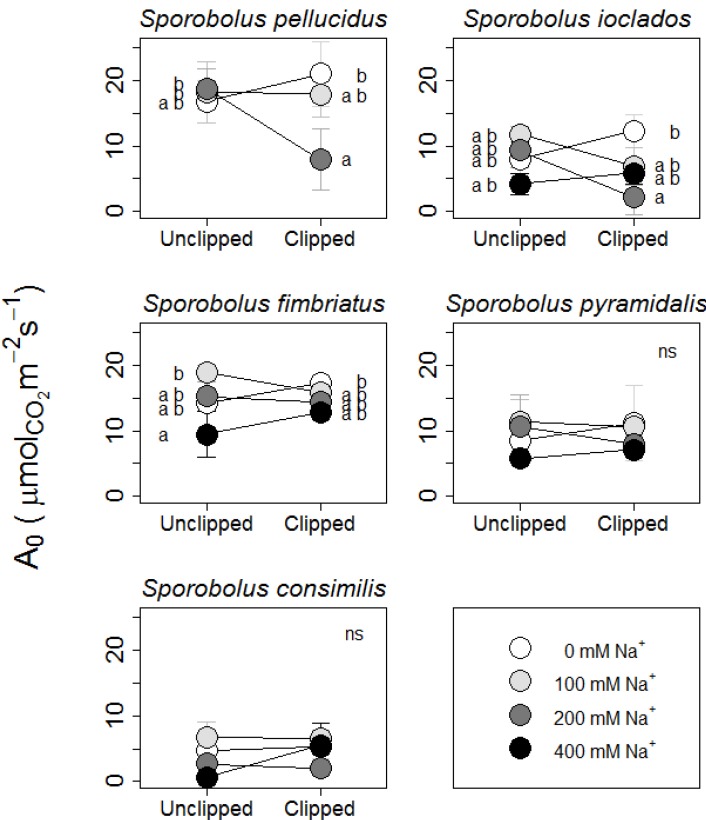
Photosynthesis at 14 days post Na and clipping treatments, represented as individual reaction norms for five *Sporobolus* species grown in factorial combination of Na (four levels) and clipping (two levels). Points are means ± SE. The point annotations (“a” and “b”) indicate statistically significant differences between groups, across all treatment combinations but within species, given by Tukey’s Honestly Significant Difference on the linear mixed effects model. Species plots are ordered by the maximum leaf height of the represented species. There are no data for *S. pellucidus* at 400 mM Na due to mortality.

A_0_ at day two was only impacted by Na; however, Na addition interacted negatively with the effects of defoliation for G_s_ and E (Table 1). Consistent with the RGR results, 100 mM Na increased photosynthesis at day two while higher treatments decreased it. All gas exchange measurements (A_0_, G_s_, and E) showed a negative interaction between Na and defoliation at day 14. For carbon assimilation at 14 days, this result stems from the large decrease in photosynthetic activity of clipped plants that were given 200 mM Na (contrast ± SE, −6.5 ± 1.4 µmol CO_2_ m^−2^ s^−1^, *p* < 0.001) when compared with 0 mM, while unclipped plants were unaffected by this level of Na addition. More specifically, clipping at 200 mM Na decreased day 14 photosynthesis in *S. pellucidus* (contrast ± SE, −13.15 ± 3.57 µmol CO_2_ m^−2^ s^−1^, *p* = 0.003; Figure 1, top left) and *S. ioclados* (contrast ± SE, −10.13 ± 3.28 µmol CO_2_ m^−2^ s^−1^, *p* = 0.042; Figure 1, top right).

### 2.2. Association between Survivorship & Traits

**Figure 2:**
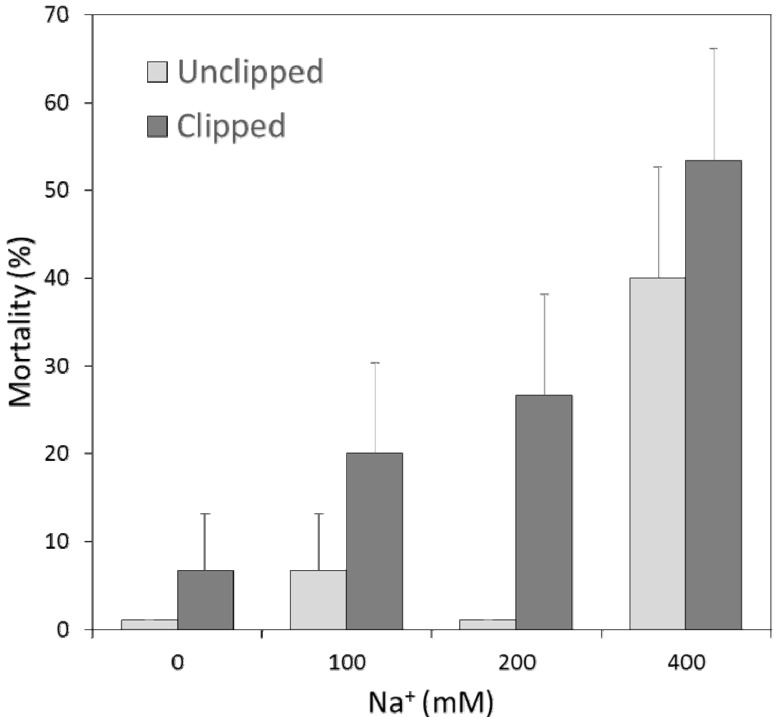
Mortality over the duration of the 10-week greenhouse experiment given by different Na concentrations and separated by defoliation treatment. Mortality is the percentage plants that died in a given group, across all species. Bars represent means ± SD of the Bernoulli distribution. Means of 0 (no error bars) have been “bumped” up to indicate the presence of data. Na and defoliation both have significant main effects in a Cox proportional hazards regression; however, the interaction effect was not significant.

In all but the highest Na treatment (400 mM Na), mortality was higher when species were exposed to the combined effects of Na and defoliation (χ^2^ = 4.03, df = 1, *p* = 0.045). Na treatment increased mortality significantly in the 400 mM treatment (contrast ± SE, 3.03 ± 1.03, *p* = 0.016; Figure 2) and defoliation increased mortality significantly (contrast ± SE, 0.94 ± 0.45, *p* = 0.039; Figure 2). Mortality was clustered in the 400 mM Na treatment and in combinations of lower Na and clipping.

**Figure 3:**
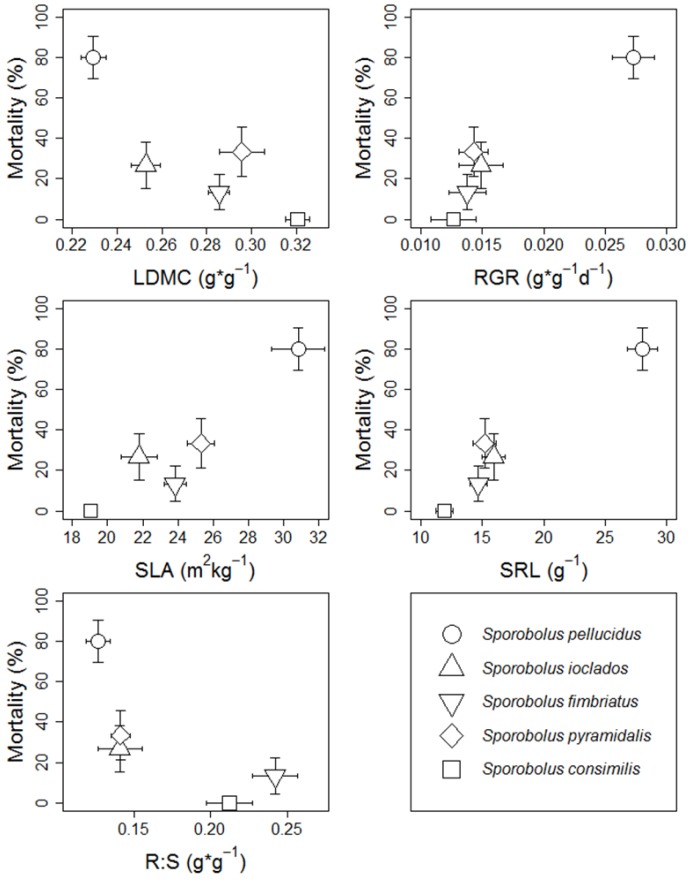
Mortality (± SD of Bernoulli distribution) plotted against the mean (± SE) trait values of five *Sporobolus* species. LDMC = leaf dry matter content; RGR = relative growth rate; SLA = specific leaf area; SRL = specific root length; and R:S = root to shoot dry mass ratio. Shapes represent different species found in the legend.

**Figure 4:**
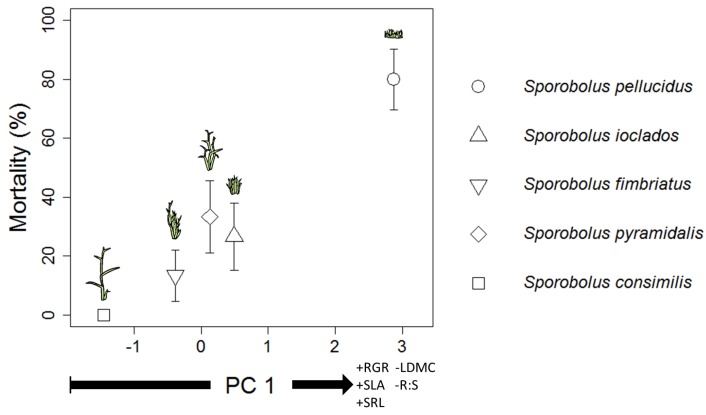
Mortality (±SD of Bernoulli distribution) plotted against PC1 from a PCA of species trait data. Shapes represent different species in the legend, each with a distinctive cartoon. The sign (+ or −) before the trait name on the x axis indicates its loading onto PC 1 (0.39, 0.48, 0.47, −0.51, −0.38 for RGR, SLA, SRL, LDMC, and R:S respectively).

Mortality was different among species and was highest in *S. pellucidus* but was lowest (none) in *S. consimilis* (χ^2^ = 24.76, df = 4, *p* < 0.001). There were significant positive correlations between mortality and RGR (*r* = 0.95, *p* = 0.026), SLA (*r* = 0.94, *p* = 0.026), and specific root length (SRL; *r* = 0.97, *p* = 0.026; Figure 3). The correlation between mortality and leaf dry matter content (LDMC) was marginal (*r* = −0.84, *p* = 0.088). Root-Shoot ratio (R:S) was not correlated with mortality (*p* = 0.15). To explore how mortality was related to the combined trait properties of the grasses we conducted a principal components analysis (PCA) using RGR, SLA, LDMC, SRL, and R:S. The first principal component (PC1) from this analysis explained 80% of the variance in the trait data. The correlation of PC1 with mortality was stronger than any trait alone (*r* = 0.98, *p* = 0.006; Figure 4).

## 3. Discussion

Due to the high demand of herbivores for Na, we expected that Na-tolerant species would also have heightened tolerance of defoliation. Consequently, our a priori hypothesis was that species would exhibit cross-tolerance between defoliation and Na stress tolerances. Previous literature suggested that short plants, such as *S. pellucidus* and *S. ioclados* (33.35 and 47.34 cm maximum greenhouse heights, respectively), would be more able to compensate for tissue loss (defoliation tolerance) relative to taller plants, such as *S. fimbriatus*, *S. pyramidalis*, and *S. consimilis* (62.98, 73.34, and 79.14 cm, respectively) [33]. Consequently, we expected *S. pellucidus* and *S. ioclados* to be relatively Na-tolerant relative to the other, taller species. Alternatively, Na tolerance represents a major adaptation of some Serengeti grasses [34] and it is plausible that species are limited in their capacity to simultaneously maximize defoliation and Na tolerance. In contrast to both expectations, we found that plants common to the heavily grazed Serengeti Plains [35], such as *S. pellucidus* and *S. ioclados* [30,36], did not have added ability to tolerate these compound stressors. Therefore, the defoliation tolerances of different *Sporobolus* species were not correlated to their Na tolerances—they were not characterized either by tradeoff or positive association between tolerances. In the context of these congeners, we concluded that Na and defoliation tolerances operate independently.

Two species abundant in the Serengeti Plains showed strong negative interaction effects of Na and defoliation on their photosynthetic activity after two weeks. These species were *S. pellucidus*, a drought tolerant plant associated with moderate Na soils in “short” and “mid-grass” sites, and *S. ioclados* which grows in “short” to “mid-grass” habitat also on moderate soil Na [36,37,38]. *S. fimbriatus* is also a “short” to “mid-grass” Plains species whereas *S. consimilis* and *S. pyramidalis* are “tall-grass” species often restricted to alkaline drainages or low Na mesic sites, respectively [31,32]. The harmful effects of our treatments where particularly adverse for the physiology of *S. pellucidus*, which had much higher mortality than the other four species when subjected to Na and defoliation. This might indicate that *S. pellucidus* would have a higher turnover rate in nature. We speculate that some grasses could have other mechanisms of compensating for these compound stressors, instead of increased tolerance [39,40]. Species might have higher fecundity, dispersal ability, or a wider range of favorable germination conditions that would provide a fitness benefit. Recent work has found important differences in the dispersal and germination strategies of Serengeti grasses [41].

We analyzed the correlation between traits and experimental mortality to explore the hypothesis that survivorship was linked to a particular life history strategy. Plant traits included LDMC (associated with resource use and leaf longevity), SLA (growth rate and herbivory tolerance), SRL (rate and nutrient uptake), and R:S (biomass allocation)—see methods [29,42]. SLA, SRL, and RGR were the strongest associated traits with mortality among species. They are highly correlated traits that represent a continuum of strategies ranging from fast growing plants with “low-cost” leaves and root to slower growing plants that adopt more conservative leaf and rooting strategies [43]. Species that senesce in high defoliation and high Na conditions appear to be associated with growth rate and high turnover. However, we do not know how two previously studied Serengeti grasses, *S. spicatus* and *S. kentrophyllus*, would have responded to our treatments [24,26]. We also don’t know how the broader species pool of Serengeti grasses would respond to our treatments. 

In general plant growth and photosynthesis were negatively impacted by Na, except at 100 mM Na. Plants may mitigate Na effects through a combination of, but not limited to, (1) compatible solute accumulation; (2) Na exclusion from the root symplast; (3) sequestration of Na in vacuoles or particular tissues; (4) secretion of Na to the leaf surface; and (5) other molecular responses to stress (reviewed in [44]). Additionally, [34] found that some East African grasses express Heat Shock Proteins (molecular chaperones) in response to Na addition. Na response generally decreases growth rate and gas exchange in plants [25]. The increase in growth and photosynthesis with low Na addition is similar to results found for related halophytes that are stimulated by adding nontoxic levels of Na, including the related C_4_ grass *Spartina alterniflora* [25].

Defoliation also reduced RGR in this experiment. RGR reductions were also found in defoliation treatments for Serengeti *Sporobolus* by [24], but defoliation is often expected to increase RGR when species are defoliation tolerant. Addition of *ad libitum* nutrients and high soil moisture in this experiment could have influenced responses to treatments. Similar interactions between defoliation and abiotic stress are influenced by drought conditions [3,45]. Finally, defoliation of plants was done as a proportion of above ground biomass so that a similar degree of defoliation stress was applied to each species (see Experimental Section). We chose this method because it allowed us to compare the physiological interaction between Na and defoliation across species. This approach differs from the commonly used method of clipping plants to a set height (e.g., 10 cm) which would impose a proportionally higher tissue loss on large plants.

Regions of high soil Na such as the Serengeti Plains are often, broadly speaking, those which experience the highest grazing pressure. Moreover, animals have high requirements for Na and phosphorus, especially during lactation, that would not be met by a diet consisting primarily of the most abundant Serengeti grass (*Themeda triandra*) [46]. This suggests that grazers may become focused on grasses that will meet their nutritional requirements (Na accumulating grasses) [47]. It is also the case that forage Na is higher in areas of high herbivore densities and that nitrogen and Na mineralization are increased in response to grazing [19,26,27]. Together, these lines of evidence not only give the impression that grazing adapted plants tolerate higher levels of Na than their less palatable counterparts, but that grazing actively increases the level of Na that they experience. Our results indicate that, despite this ecological setting, the *Sporobolus* species studied here do not have any additional ability to tolerate the combined effects of Na and herbivory. Furthermore, the Na and defoliation tolerances of species were independent of each other. Belsky [21,22,36] suggested that in the Serengeti Plains, mosaic plant communities might be explained in part by Na and, in general, soil Na was a strong predictor of vegetation heterogeneity. The Serengeti grasses in our experiment differ in their responses to Na when defoliated, and those differences are likely important in generating the patterns of species diversity found in areas of high Na and grazing.

## 4. Experimental Section

### 4.1. Plant Material and Greenhouse

Three genotypes each of five *Sporobolus* species (*S. consimilis*, *S. pyrimidalis*, *S. fimbriatus*, *S. ioclades*, and *S. pellucidus*) were propagated clonally from laboratory-grown populations originally sourced from different areas in their ranges in Serengeti National Park (2°19'58''N, 34°34'00''E; Tanzania). The experiment was conducted in a greenhouse at Wake Forest University (~300–1400 µmol m^−2^ s^−1^ PAR and ~28 °C midday). Before beginning the treatment phase, 5 connected tillers were selected for experimentation. Another 5 tillers were subsampled to get an estimate of starting dry weight, via the wet to dry mass ratio of each individual plant. All plants were clipped to 10 cm at the roots and to 50% of their shoot biomass (see Section 4.2). Plants were grown in a 3:1:1 mixture of 360 Metromix soil with sand and perlite in 10 × 30 cm PVC pots. Osmocote nutrient pellets were added to this soil mixture in a volumetric ratio of 1:40. Soils were kept at 80% of water holding capacity by watering to a set mass every ~2 days. In total, 120 plants were grown in factorial treatment of Na^+^ and defoliation for 10 weeks (May–July 2011). The experimental design was 4 Na levels × 2 defoliation levels × 5 species × 3 clones.

### 4.2. Treatments

Individuals were subjected to one of four different soil Na (Na^+^) dosage levels (0, 100, 200, and 400 mM). Na was added in two pulses (weeks 1 and 5) as an equimolar solution of NaCl and NaHCO_3_ and monitored with a Spectrum Field Scout EC probe. Measured electrical conductivity was used to estimate actual Na^+^ concentration through calibration with samples analyzed by ICP (Varian Vista AX, Mulgrave, Australia). The r^2^ value for the relationship between observed and expected soil Na values is 0.995. Na application concentration and techniques have been modified from other studies [24,34] and are intended to simulate early wet season conditions when rainfall brings soil Na^+^ into solution.

Clipping consisted of two levels, no defoliation and removal of 50% of above ground biomass of each individual at weeks one and three. The proportional height at which each species should be clipped to remove 50% of the above ground biomass was determined in a pilot study. This treatment introduces a tissue removal without creating a bias against large statured plants with fewer thicker tillers. It also allows us to comparatively test specifically for relative physiological effects of defoliation rather than advantageous differences in plant stature.

### 4.3. Measurements

Leaf gas exchange was measured two days and 14 days after treatment initiation. Measurements of A_o_ (net carbon assimilation; µmol CO_2_ m^−2^ s^−1^), G_s_ (stomatal conductance; mol H_2_O m^−2^ s^−1^), and E (transpiration; mmol H_2_O m^−2^ s^−1^) were made with a Li-cor 6400 on the youngest fully expanded leaf. Plants were kept in a climate controlled chamber (~30 °C and ~800 µmol m^−2^ s^−1^) for 1 h. prior to making gas exchange measurements.

Final plant biomass was quantified by drying and weighing all above and belowground plant tissue at the end of the experiment. Terminal sampling of biomass included removal of soil, drying of whole plants at 65 °C to constant weight, and the division and separate weighing of plant roots, crowns, and shoots. RGR was calculated as the difference in the natural logs of the final dry mass and initial estimated plant dry mass divided by time. The root to shoot ratio (R:S) was calculated as the ratio of total dry weight of all belowground plant parts to that of aboveground and clipped biomass.

A variety of functional traits (LDMC, SLA, SRL, and R:S) were measured with standard methods [34,43]. At the end of the experiment, three leaf subsamples were taken from each plant and rehydrated overnight in diH_2_O at 4 °C [42]. Each subsample was pressed between glass, and its area was estimated from a photo (SigmaScan; Systat Software, San Jose, CA, USA). Subsamples were blotted dry, weighted, dried at 65 °C to constant weight, and then reweighted. LDMC was taken as the ratio of dry to wet weight for each plant. SLA was taken as the ratio of measured area to dry weight. Roots were pressed between sheets of glass and imaged for total length at the end of the experiment and then divided by the dry weight of the roots to calculate SRL [48].

### 4.4. Statistical Analysis

Statistics were conducted in R [49]. Linear mixed-effects models were used to test for effects of Na addition, defoliation, and Na by defoliation interaction effects on RGR, gas exchange parameters, and all functional traits using plant clone as a nested random effect within species. Individual model selection and evaluation of model assumptions were conducted following [50] and using the R package “nlme” [51]. Post-hoc comparisons (α = 0.05) were conducted using the “multcomp” package [52]. Because of high mortality, we conducted survivorship analysis. Survivorship was modeled with Cox proportional hazards regression using the package “survival” [53]. Because of sample size the Cox regression models would not converge when interaction terms were included; therefore, we used a Cochran-Mantel-Haenszel test to assess the independence of Na and defoliation with respect to mortality (“vcd” R package) [54]. To understand whether certain combinations of plant traits tended to be associated with survivorship, correspondence of mortality and all plant functional traits were assessed at the species level using Pearson correlation. False Discovery Rate correction was used to correct for the use of multiple tests and we present FDR-adjusted *p*-values, or “*q*-values” [55,56]. Finally, principal component analysis was conducted on the species mean trait values (LDMC, RGR, SLA, SRL, and R:S) with R [49]. Pearson correlation was used on PC1 and species mortality to gauge the degree of association between mortality and the primary axis of species trait variation. 

## 5. Conclusions

In this study, we examined the growth and physiology of five congeners in response to combinations of soil Na and defoliation in the same controlled experiment. In contrast to our predictions, species tolerances to Na and defoliation where neither positively correlated nor indicative of a tradeoff—they were independent of each other among species. Instead, species from areas of high grazing intensity were adversely affected by the combination of Na and herbivory, resulting in high mortality. This suggests that species, such as *S. pellucidus* and *S. ioclados* in particular, may have high turnover rates in nature. As such, we speculate that species may compensate through increased fecundity, dispersal, or range of germination conditions. Because species had different responses to these stressors, the combined effects of defoliation and Na are likely to be important for understanding coexistence of these species and the evolutionary patterns of Na and defoliation tolerances [25,57].

## Appendix

**Table A1:** Companion table to Table 1 in text, showing *F*, df, and *p* values for the full linear mixed model results. Blank values indicate interaction or no significance.

Response	Na^+^ treatment			Defoliation			Na^+^ x Defoliation		
	*F*	df	*p*	*F*	df	*p*	*F*	df	*p*
RGR	8.71	3,78	<0.0001	84.86	1,78	<0.0001			
*Day 2*									
A_0_	31.25	3,102	<0.0001						
Gs							3.09	3,98	0.0306
E							2.85	3,98	0.0414
*Day 14*									
A_0_							6.77	3,88	0.0004
Gs							5.77	3,88	0.0012
E							5.54	3,88	0.0016
